# Supplementary material for: An efficient and accurate distributed learning algorithm for modeling multi-site zero-inflated count outcomes
Source: Sci Rep. 2021 Oct 4;11:19647. doi: 10.1038/s41598-021-99078-2 (PMC8490431; doi:10.1038/s41598-021-99078-2)
Supplement: Supplementary file 1 — Supplementary Information. [file 41598_2021_99078_MOESM1_ESM.pdf]

# Supplementary Material

An efficient and accurate distributed learning algorithm for modeling multi-site zero-inflated count outcomes

Mackenzie J. Edmondson[a], Chongliang Luo[a], Rui Duan[b], Mitchell Maltenfort[c], Zhaoyi Chen[d,e], Kenneth Locke Jr.[a], Justine Shults[a], Jiang Bian[d,e], Patrick B. Ryan[f], Christopher B. Forrest[c], Yong Chen[a]

a. Department of Biostatistics, Epidemiology, and Informatics, University of Pennsylvania Perelman School of Medicine, Philadelphia, PA, USA
b. Department of Biostatistics, Harvard T.H. Chan School of Public Health, Boston, MA, USA
c. Department of Pediatrics, Children's Hospital of Philadelphia, Philadelphia, PA, USA
d. Department of Health Outcomes and Biomedical Informatics, College of Medicine, University of Florida, Gainesville, FL, USA
e. Cancer Informatics Shared Resource, University of Florida Health Cancer Center, Gainesville, FL, USA
f. Janssen Research and Development, Titusville, NJ, USA

S.1) First-and second-order gradient formulations

**Logistic component:**

$$\nabla l_1(\boldsymbol{\beta}) = \frac{\partial}{\partial \beta} l_1(\boldsymbol{\beta}) = \sum_{i=1}^{n} \boldsymbol{X}_i - \sum_{i=1}^{n} \frac{\exp(\boldsymbol{X}_i^t \boldsymbol{\beta})}{1 + \exp(\boldsymbol{X}_i^T \boldsymbol{\beta})} \boldsymbol{X}_i \qquad (\mathrm{S.1})$$

$$\nabla^2 l_1(\boldsymbol{\beta}) = \frac{\partial^2}{\partial \boldsymbol{\beta} \partial \boldsymbol{\beta}^T} l_1(\boldsymbol{\beta}) = -\sum_{i=1}^{n} \frac{\exp(\boldsymbol{X}_i^T \boldsymbol{\beta})}{(1 + \exp(\boldsymbol{X}_i^T \boldsymbol{\beta}))^2} \boldsymbol{X}_i \boldsymbol{X}_i^T \qquad (\mathrm{S.2})$$

**Zero-truncated Poisson component:**

$$\nabla l_2(\boldsymbol{\gamma}) = \frac{\partial}{\partial \boldsymbol{\gamma}} l_2(\boldsymbol{\gamma}) = \sum_{Y_i > 0} \left( -\exp(\boldsymbol{Z}_i^T \boldsymbol{\gamma}) \, \boldsymbol{Z}_i + y_i \boldsymbol{Z}_i - \frac{\exp(-\exp(\boldsymbol{Z}_i^T \boldsymbol{\gamma})) \exp(\boldsymbol{Z}_i^T \boldsymbol{\gamma}) \, \boldsymbol{Z}_i}{1 - \exp(-\exp(\boldsymbol{Z}_i^T \boldsymbol{\gamma}))} \right) \qquad (\mathrm{S.3})$$

$$\nabla^2 l_2(\boldsymbol{\gamma}) = \frac{\partial^2}{\partial \boldsymbol{\gamma} \partial \boldsymbol{\gamma}^T} l_2(\boldsymbol{\gamma}) = \sum_{Y_i > 0} -\exp\left(\boldsymbol{Z}_i^T \boldsymbol{\gamma}\right) \boldsymbol{Z}_i \boldsymbol{Z}_i^T + \sum_{Y_i > 0} \left( \frac{\exp\left(\boldsymbol{Z}_i^T \boldsymbol{\gamma}\right) \left[\exp\left(\exp\left(\boldsymbol{Z}_i^T \boldsymbol{\gamma}\right) + \boldsymbol{Z}_i^T \boldsymbol{\gamma}\right) - \exp\left(\exp\left(\boldsymbol{Z}_i^T \boldsymbol{\gamma}\right)\right) + 1\right]}{\left[\exp\left(\exp\left(\boldsymbol{Z}_i^T \boldsymbol{\gamma}\right)\right) - 1\right]^2} \boldsymbol{Z}_i \boldsymbol{Z}_i^T \right) \qquad (\mathrm{S.4})$$

## S.2) Additional simulation results

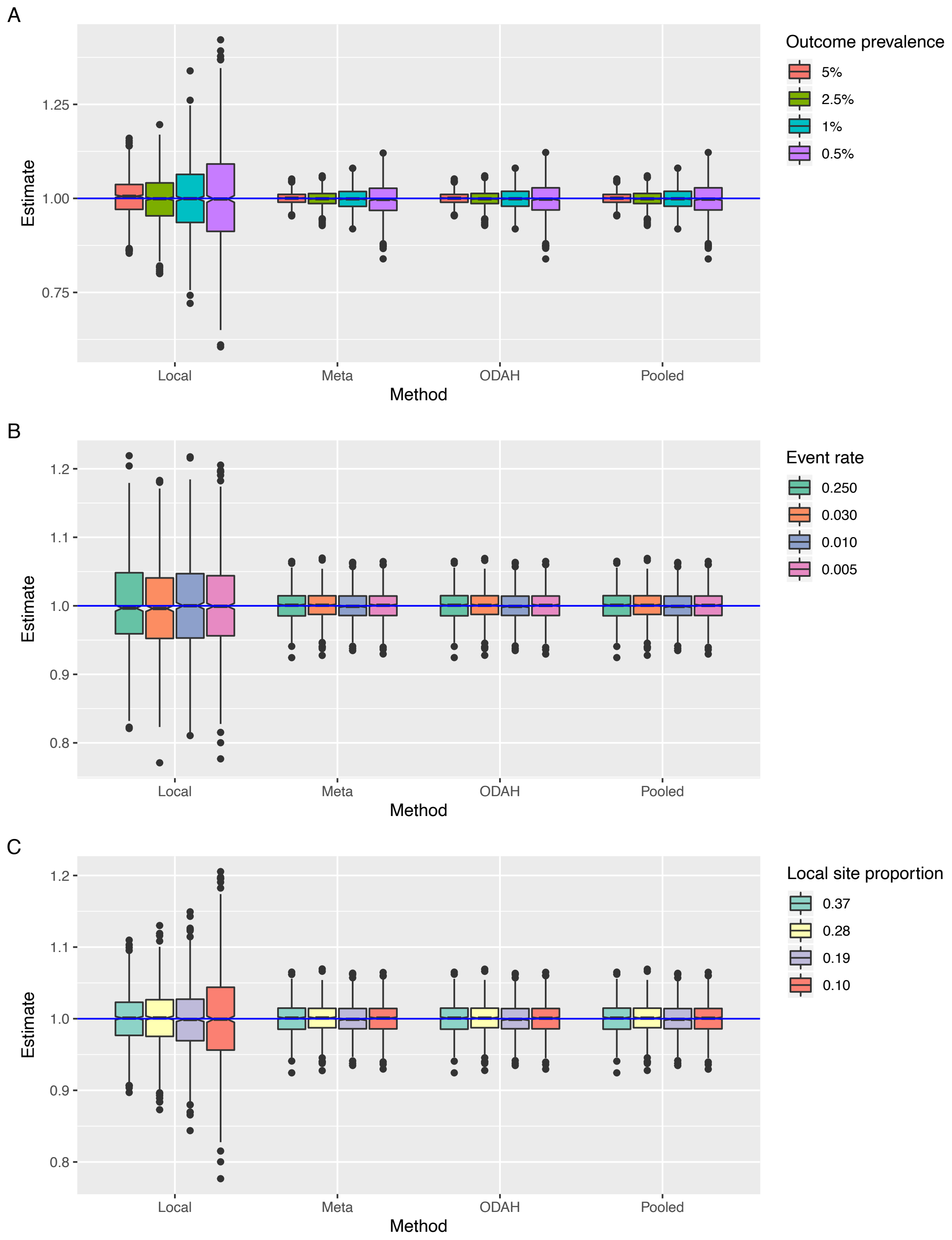


Figure S.2. Simulation results for estimating logistic component covariate $\beta_2$. A) Results for Setting A, fixing $n_{local}$ = 20,000 and $\gamma_0$ = -3.6 ($\lambda = 0.03$) while varying outcome prevalence. B) Results for Setting B, fixing $n_{local}$ = 20,000 and $\beta_0$ = -3.7 (2.5% prevalence) while varying event rate ($\lambda$). C) Results for Setting C, fixing $\beta_0$ = -3.7 (2.5% prevalence) and $\gamma_0 = -3.6$ ($\lambda = 0.03$) while varying proportion of observations in local site. Horizontal blue line represents true value of $\beta_2 = 1$.
